# Rapid, dose-dependent and efficient inactivation of surface dried SARS-CoV-2 by 254 nm UV-C irradiation

**DOI:** 10.2807/1560-7917.ES.2021.26.42.2001718

**Published:** 2021-10-21

**Authors:** Natalia Ruetalo, Ramona Businger, Michael Schindler

**Affiliations:** 1 Institute for Medical Virology and Epidemiology of Viral Diseases, University Hospital Tübingen, Tübingen, Germany

**Keywords:** SARS-CoV-2, UV-C irradiation, decontamination, disinfection, infection control, COVID-19

## Abstract

Background: The COVID-19 pandemic urges for cheap, reliable, and rapid technologies for disinfection and decontamination. One frequently proposed method is ultraviolet (UV)-C irradiation. UV-C doses necessary to achieve inactivation of high-titre SARS-CoV-2 are poorly defined.

Aim: We investigated whether short exposure of SARS-CoV-2 to UV-C irradiation sufficiently reduces viral infectivity and doses necessary to achieve an at least 6-log reduction in viral titres.

Methods: Using a box and two handheld systems designed to decontaminate objects and surfaces, we evaluated the efficacy of 254 nm UV-C treatment to inactivate surface dried high-titre SARS-CoV-2.

Results: Drying for 2 hours did not have a major impact on the infectivity of SARS-CoV-2, indicating that exhaled virus in droplets or aerosols stays infectious on surfaces for at least a certain amount of time. Short exposure of high titre surface dried virus (3–5*10^6 IU/ml) with UV-C light (16 mJ/cm^2^) resulted in a total inactivation of SARS-CoV-2. Dose-dependency experiments revealed that 3.5 mJ/cm^2^ were still effective to achieve a > 6-log reduction in viral titres, whereas 1.75 mJ/cm^2^ lowered infectivity only by one order of magnitude.

Conclusions: SARS-CoV-2 is rapidly inactivated by relatively low doses of UV-C irradiation and the relationship between UV-C dose and log-viral titre reduction of surface residing SARS-CoV-2 is nonlinear. Our findings emphasize that it is necessary to assure sufficient and complete exposure of all relevant areas by integrated UV-C doses of at least 3.5 mJ/cm^2^ at 254 nm. Altogether, UV-C treatment is an effective non-chemical option to decontaminate surfaces from high-titre infectious SARS-CoV-2.

## Introduction

The severe acute respiratory syndrome coronavirus-2 (SARS-CoV-2) has spread globally since January 2020 and there is an urgent need for rapid, highly efficient, environmentally friendly, and non-chemical disinfection procedures. Application of ultraviolet (UV)-C light is an established technology for decontamination of surfaces and aerosols [1-3]. This procedure has proven effective to inactivate SARS-CoV-1 [4-6], several other enveloped and non-enveloped viruses as well as bacteria [7]. UV-C-based disinfection could be applied in operating rooms and healthcare facilities and it also proved useful in the business sector, where it is necessary to sterilise surfaces frequently touched by multiple individuals. Some examples discussed in the context of public health are escalators, public transportation, rental cars, door handles and waiting rooms.

Recently, it has also been shown that SARS-CoV-2 is sensitive to inactivation by UV-C irradiation [8-12]. However, some of the studies used high UV-C doses from 108 mJ/cm^2^ to more than 1 J/cm^2^ at exposure times from 50 s to several minutes for total inactivation of SARS-CoV-2 [10-12]. These parameters are in a range complicating efficient application of UV-based methods for large-scale decontamination of surfaces and aerosols. Other studies used innovative 222 nm or 280 nm UV-C light-emitting diode (LED) technologies [8,9] which are not yet implemented in most established 254 nm UV-C-based decontamination devices and needed relatively high doses of UV-C irradiation for inactivation. Another recent study by Storm et al. established 254 nm UV-C dose-dependency inactivation kinetics of SARS-CoV-2 and reported doses necessary for complete sterilisation of dry and wet virus preparations between 4 s and 9 s at 0.85 mW/cm^2^ in a test box [13]. While these data are promising, a limitation of the study design was the use of a test box and relatively low viral titres which allowed for only 2- to 3- log titre reductions by the treatment.

The exact knowledge about dose-dependent inactivation kinetics is essential to design UV-C-based decontamination procedures that allow definite disinfection of SARS-CoV-2. We hence conducted an approach simulating the inactivation of dried surface residing high-titre infectious SARS-CoV-2 by two mobile handheld (HH) UV-C emitting devices and an UV-C box designed to decontaminate medium-size objects. We investigated whether short exposure of SARS-CoV-2 to UV-C irradiation is sufficient to reduce viral infectivity and which UV-C doses are necessary to achieve an at least 6-log reduction in viral titres.

## Methods

### Cell culture

Caco-2 (human colorectal adenocarcinoma) cells were cultured at 37 °C with 5% CO_2_ in Dulbecco's Modified Eagle Medium (DMEM) containing 10% fetal calf serum (FCS), with 2 mM l-glutamine, 100 μg/ml penicillin-streptomycin and 1% non-essential amino acids (NEAA).

### Viruses

The recombinant SARS-CoV-2 expressing mNeonGreen (icSARS-CoV-2-mNG) [14] was obtained from the World Reference Center for Emerging Viruses and Arboviruses (WRCEVA) at the University of Texas Medical Branch (UTMB, Galveston, United States (US)). To generate icSARS-CoV-2-mNG stocks, 200,000 Caco-2 cells were infected with 50 μl of virus stock in a 6-well plate, the supernatant was harvested 48 hours post infection (hpi), centrifuged, and stored at -80 °C. For multiplicity of infection (MOI) determination, a titration using serial dilutions of the virus stock was conducted. The number of infectious virus particles per ml was calculated as the (MOI × cell number)∕(infection volume), where MOI =  − ln(1 − infection rate).

### Ultraviolet-C light inactivation treatment

A total of 35 μl of virus stock, corresponding to ca 4–6*10^6^ infectious units (IU) of icSARS-CoV-2-mNG were spotted (in triplicates) in 6-well plates and dried for 2 hours at room temperature (RT). This setup was chosen to mimic the situation in which an infected person exhales droplets that dry on surfaces and potentially stay infectious and hazardous over a prolonged period of time. Six-well plates spotted with dried virus were treated with UV-C-light (254 nm) using the Soluva pro UV Disinfection Chamber (Heraeus, Hanau, Germany) for 60 s or the Soluva Zone HP Disinfection Handheld (Heraeus) for 2 s in a fixed regime at 5 and 20 cm plate distance. In addition, a moving regime using slow (3.75 cm/s) and fast (12 cm/s) speed at 20 cm distance was tested.

We also employed a second-generation Disinfection Handheld Soluva Zone H (Heraeus) which is less powerful than the Soluva pro UV but works autonomously with a rechargeable battery. The spectrum of UV-C lamps employed in these devices are shown in Supplemental figure 1. The lower UV-C intensity emitted by this device allowed us to perform a dose-dependency experiment exposing dried virus with different UV-C intensities. The time-dependent UV-C intensity emitted by the Soluva Zone H at various distances is detailed and depicted in Supplemental figure 2. UV-C exposure was carried out after 10 min of pre-heating the device at a distance of 50 cm for 20 s, 10 s, 5 s, 2.5 s, 20 s + 97% UV-filter, 10 s + 97% UV-filter corresponding to 14 mJ/cm^2^, 7 mJ/cm^2^, 3.5 mJ/cm^2^, 1.75 mJ/cm^2^, 0.42 mJ/cm^2^ and 0.21 mJ/cm^2^. These values are based on an on-site and parallel measurement of UV-C intensity emitted by the device via an UV-C dosimeter (Dr Gröbel UV electronic GmbH, Ettlingen, Germany), which corresponds to 0.7 mJ/cm^2^ when the UV-C light is applied at 50 cm distance, and fits well to the previously company-measured value of 0.84 mJ/cm^2^ (Supplemental figure 2).

As control, 6-well plates were spotted with the virus and dried, but not UV-C treated. After UV-C treatment, the spotted virus was reconstituted using 1 ml of infection media (culture media with 5% FCS) and viral titres determined as explained below. As additional control, 35 μl of the original virus stock were diluted to 1 ml with infection media and used as virus stock infection control. All UV-C treatments were done at RT.

### Evaluation of ultraviolet-C treatment

For infection experiments and titre determination, 1 x 10^4^ Caco-2 cells/well were seeded in 96-well plates the day before infection. Cells were incubated with the SARS-CoV-2 strain icSARS-CoV-2-mNG at a MOI = 1.1 (stock) or the UV-C treated and reconstituted virus in serial twofold dilutions from 1:200 up to 1:51,200 and in one experiment up to 1:102,400. At 48 hpi, cells were fixed with 2% paraformaldehyde (PFA) and stained with Hoechst 33342 (Thermo Fisher, Waltham, US) (1 µg/ml final concentration) for 10 min at 37 °C. The staining solution was removed and exchanged for phosphate-buffered saline (PBS). For quantification of infection rates, images were taken with the Cytation3 (Biotek, Winooski, US) and Hoechst + and mNG + cells were automatically counted by the Gen5 Software (Biotek). Viral titres i.e. the number of infectious virus particles per ml, were calculated as the (MOI × cell number)∕(infection volume), where MOI =  − ln(1 − infection rate). Infection rates lower than 0.01 were used as a cut-off and set to 0 in order to avoid false positive calculations.

### Software and statistical analysis

Experiments were repeated two to four times each, using duplicate or triplicate infections. GraphPad Prism 8.0 was used for statistical analyses (one-way ANOVA with multiple comparison and Fishers least significant difference (LSD)-test) and to generate graphs, as well as CorelDrawX7. Other software used included Gen5 v.3.10.

### Ethical statement

This study does not include any data obtained with primary patient cells or data. Hence, there was no necessity to obtain ethical approval by the internal review board.

## Results

### Inactivation of high-titre SARS-CoV-2 by ultraviolet-C treatment

**Figure 1:**
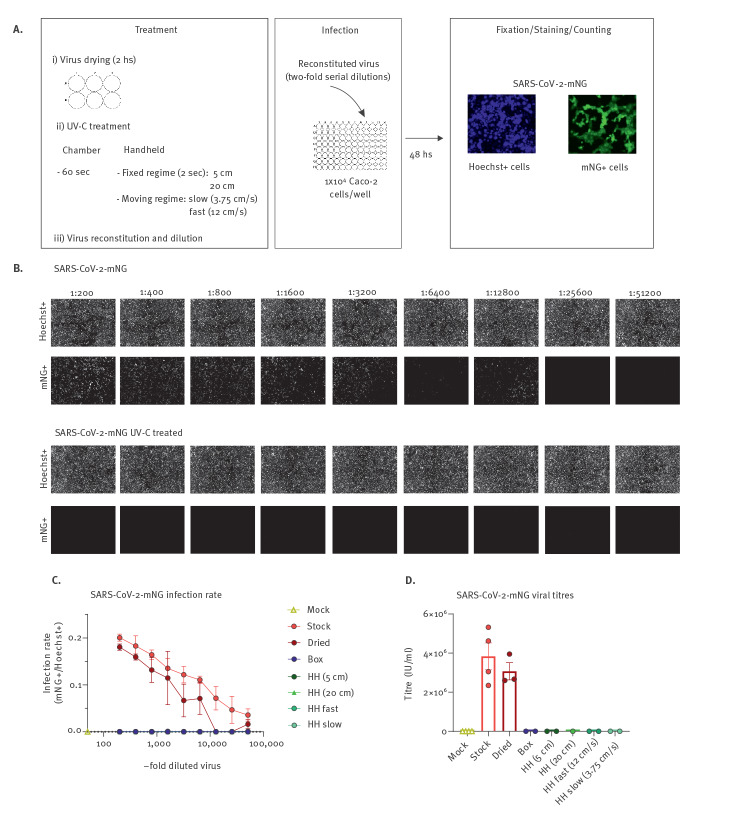
Inactivation of SARS-CoV-2 by UV-C light treatment

HH: handheld; mNG: mNeonGreen; SARS-CoV-2: severe acute respiratory syndrome coronavirus; SEM: Standard error of the mean; UV: ultraviolet; IU: infectious units.

Simulating the situation in which exhaled droplets or aerosols from infected individuals contaminate surfaces, we produced a high-titre SARS-CoV-2 infectious stock and dried 35µl of this stock corresponding to ca 4–6*10^6^ IU/ml in each well of a 6-well plate. The plates were then either non-treated or exposed to five UV-C regimens at 254 nm (Figure 1a). These include inactivation for 60 s in a box designed to disinfect medium-size objects, 2 s exposure at 5 cm or 20 cm distance with a HH UV-C disinfection device and an approach simulating decontamination of surfaces via the HH UV-C device (Zone HP). We performed this simulated HH decontamination in slow- and fast-moving speeds at a distance of ca 20 cm (Supplemental movie 1 and 2). UV-C irradiance (254 nm) in the box with an exposure time of 60 s corresponds to an irradiation dose of 600 mJ/cm^2^; for the HH device, at 5 cm the UV-C dose at 2 s irradiation time is 80 mJ/cm^2^ and at 20 cm is 16 mJ/cm^2^. From the speed of the slow and fast moving regimens we calculate a UV-C dose of 2.13 mJ/cm^2^ and 0.66 mJ/cm^2^, respectively, assuming a focused intensity beam. However, taking into consideration the UV-C light distribution underneath the HH device, the integrated UV-C dose accumulates to 20 mJ/cm^2^ for the fast regimen.

A. Experimental layout of the different UV-C treatments and the infection assay employed using the green-fluorescent virus SARS-CoV-2-mNG. B. Primary data showing the results of the infection assay using the non-treated stock virus as a positive control and the UV-C treated virus (HH, fast-moving regime). In the upper row, the total amount of cells for each well of the twofold serial dilution of virus is shown as Hoechst +. In the lower, infected cells are visualised indicated as mNG + cells. C. Infection rate curves for UV-C irradiated SARS-CoV-2-mNG using different UV-C treatments. The graph shows the infection rate at each twofold serial dilution, calculated as the number of infected cells (mNG +) over the total number of cells (Hoechst +) for the non-treated viral stock (n = 4), dried viral stock (n = 3), and dried and UV-C irradiated virus using five different UV-C treatments (n = 2). Data are presented as mean +/ − SEM with the number of biological replicates indicated above. D. SARS-CoV-2-mNG viral titres after UV-C treatment and reconstitution. The graph shows the viral titres calculated in IU/mL for the mock-infected, non-treated, and dried stock as well as the dried and UV-C irradiated virus under the different treatments. The number of biological replicates (n = 2–4) is directly plotted and indicated in 1c. Data are presented as mean +/ − SEM.

In the context of the moving fast regimen, even short UV-C treatment of the dried virus completely inactivated SARS-CoV-2. This was evident, as no infected cells were detected, when the dried virus was reconstituted in media and used to inoculate the naïve Caco-2 cells (Figure 1b). Titration of twofold series dilutions of the UV-C treated and non-treated control samples, as well as the freshly thawed strain as reference, revealed that (i) drying for 2 hours does not have a major impact on the infectivity of SARS-CoV-2 and (ii) all five UV-C treatment regimens effectively inactivate SARS-CoV-2 (Figure 1c). Calculation of viral titres based on the titration of the reconstituted virus stocks revealed a loss of titre because of drying from ca 4*10^6^ to ca 3*10^6^ IU/ml in this set of experiments and effective 6-log titre reduction of SARS-CoV-2 by all employed UV-C treatment regimens down to 16 mJ/cm^2^ (Figure 1d).

### Dose-dependent ultraviolet-C mediated inactivation of SARS-CoV-2

**Figure 2:**
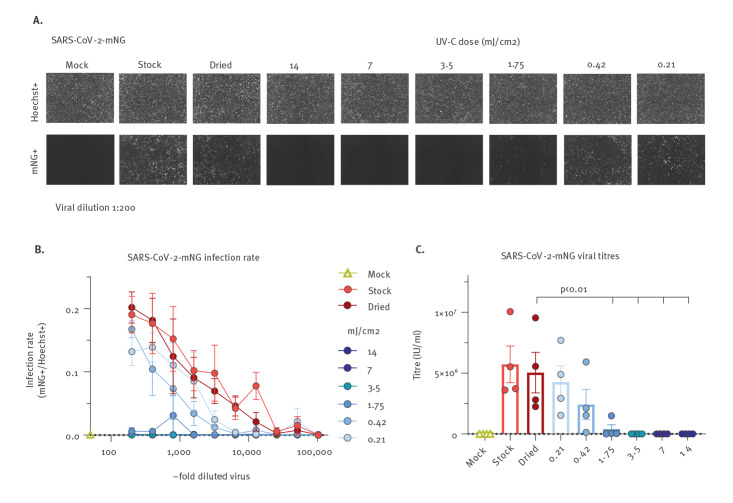
UV-C dose required for SARS-CoV-2 inactivation

ANOVA: analysis of variance; HH: handheld; LSD: least significant difference; mNG: mNeonGreen; SARS-CoV-2: severe acute respiratory syndrome coronavirus; SEM: Standard error of the mean; UV: ultraviolet; IU: infectious units.

We next aimed to determine the UV-C doses at 254 nm that are sufficient to achieve complete disinfection with an at least 6-log reduction in viral titres. For this, we employed a battery-driven UV-C HH device (Zone H) emitting 254 nm UV-C light at 0.7 mJ/cm^2^ at a distance of 50 cm. This allowed us to treat surface dried SARS-CoV-2 with different UV-C doses by variation of the exposure time and additional use of a 97% UV-C filter. In agreement with our previous measurement, drying for 2 hours did not considerably affect SARS-CoV-2 infectivity and relatively high doses of 254 nm UV-C treatment (14 mJ/cm^2^) inactivated SARS-CoV-2 (Figure 2a exemplary images at 1:200 dilution and Figure 2b quantitative analyses). Furthermore, there was a dose-dependent reduction in SARS-CoV-2 infectivity with total inactivation down to 3.5 mJ/cm^2^ while partial inactivation was still observed at 1.75 mJ/cm^2^ (Figure 2a and b). Careful evaluation of viral titres post UV-C exposure revealed that > 6-log titre reduction was achieved by 3.5 mJ/cm^2^ 254 nm UV-C treatment (Figure 2c). Of note, mean titres were only reduced by slightly more than one order of magnitude from 5.04*10^6^ IU/ml of the dried and reconstituted SARS-CoV-2 to 3.5*10^5^ IU/ml when the virus was exposed to 1.75 mJ/cm^2^, corresponding to 93% inactivation. Therefore, the relationship between inactivation of surface dried SARS-CoV-2 and UV-C treatment is nonlinear, at least in our system, and 3.5 mJ/cm^2^ are necessary to achieve a 6-log titre reduction.

A. Primary data showing the results of the infection assay using mock-infected cells, non-treated stock virus as a positive control, and virus treated with the 6 UV-C doses as indicated. In the upper row, the total amount of cells is shown as Hoechst +. In the lower, infected cells at a viral dilution of 1:200 are visualised indicated as mNG + cells. B. Infection rate curves for UV-C irradiated SARS-CoV-2-mNG using different UV-C doses. The graph shows the infection rate at each twofold serial dilution, calculated as the number of infected cells (mNG +) over the total number of cells (Hoechst +) for the non-treated viral stock, dried viral stock, and dried and UV-C irradiated virus using different UV-C-doses (n = 4). Data are presented as mean +/ − SEM with the number of biological replicates indicated above. C. SARS-CoV-2-mNG viral titres after UV-C treatment. The graph shows the viral titres calculated in IU/mL for the mock-infected, non-treated, and dried stock as well as the dried and UV-C irradiated virus under the different UV-C-doses. The number of biological replicates is n = 4. Data are presented as mean +/ − SEM. For analysis of statistical significance, we used a one-way ANOVA with multiple comparison and Fishers LSD-test.

## Discussion

Disinfection of surfaces and aerosols by UV-C irradiation is an established, safe and non-chemical procedure used for the environmental control of pathogens [1-3,15]. UV-C treatment has proven effective against several viruses including SARS-CoV-1 [4-6] and other coronaviruses, i.e. canine coronaviruses [16]. Hence, as recently demonstrated by others [8-13] and now confirmed by our study, it was expected that SARS-CoV-2 can be inactivated by UV-C treatment.

One critical question is the suitability of this technology in a setting in which the exposure time of surfaces or aerosols should be kept as short as possible to allow for a realistic application, such as in rooms that need to be used frequently as operating rooms or lecture halls. In such settings, we assume that the virus is exhaled from an infected person by droplets and/or aerosols, dries on surfaces and hence represents a threat to non-infected individuals. We mimicked such a situation and first evaluated if surface dried SARS-CoV-2 is infectious. Drying for 2 hours, in agreement with previous work [13,17], did not result in a significant reduction of viral infectivity, indicating smear-infections could indeed play a role in the transmission of SARS-CoV-2. On the other hand, our virus-preparations are dried in cell culture pH-buffered medium containing FCS, which might stabilise viral particles. Hence, even though this is not the scope of the current study, it will be interesting to evaluate if longer drying or virus-preparations in PBS affect the environmental stability of SARS-CoV-2. Irrespective of the latter, UV-C-exposure of dried high-titre SARS-CoV-2 preparations containing ca 3–5*10^6^ IU/ml after reconstitution resulted in a complete reduction of viral infectivity. In this context, it is noteworthy that we achieved a 6-log virus-titre reduction in a setting simulating surface disinfection with a moving handheld device. With our fast-moving protocol, the calculated integrated UV-C dose of 20 mJ/cm^2^ at 254 nm, was substantially lower than the previously reported 1,048 mJ/cm^2^ necessary to achieve a 6-log reduction in virus titres when exposing aqueous SARS-CoV-2 to UV-C [10]. In another study using a 222 nm UV-LED source, 3 mJ/cm^2^ lead to a 2.51-log (99.7%) reduction of infectious SARS-CoV-2 when irradiating for 30 s; however, inactivation did not increase with extended irradiation regimens up to 300 s [9]. In addition, 20 s deep-ultraviolet treatment at 280 nm corresponding to a dose of 75 mJ/cm^2^ reduced SARS-CoV-2 titre up to 3-logs [8]. Finally, Storm and colleagues reported a 2-log reduction of dried SARS-CoV-2 at 4 s with 0.85 mW/cm^2^ corresponding to 3.4 mJ/cm^2^ [13]. Of note, this value is highly similar to the dose of 3.5 mJ/cm^2^ calculated by us to be sufficient to achieve a > 6-log SARS-CoV-2 titre reduction when the virus is in a dried surface residing state (Figure 2). Comparing these values to other pathogens, SARS-CoV-2 seems particularly sensitive towards UV-C light. To achieve a 3-log titre reduction, 75–130 mJ/cm^2^ are necessary for adenovirus, 11–28 mJ/cm^2^ for poliovirus, and bacteria such as *Bacillus subtilis* require 18–61 mJ/cm^2^ [7].

Important limitations of UV-C-based disinfection procedures exist. First and most importantly, UV-C irradiation is harmful to humans because of the high energy of the germicidal lamps and exposure of skin or eyes must be avoided. This excludes decontamination of populated public spaces by UV-C. Furthermore, UV-C does not penetrate surfaces, hence for efficient disinfection, equal direct irradiation of all surfaces with a sufficient dose has to be assured. Our work highlights this aspect, as due to the nonlinear decay kinetic of the dose-response relationship, 3.5 mJ/cm^2^ will totally inactivate high viral titres, whereas a slightly reduced dose of 1.75 mJ/cm^2^ only achieves roughly one-log reduction (Figure 2c).

Apart from that, our study, as well as the research done by others [13], emphasises UV-C-based disinfection technologies as highly efficient to rapidly sterilise surfaces in different settings such as operating rooms, less-frequently populated areas in healthcare facilities and public transportation, as well as in research facilities. Ideally, applications should be performed in closed containers, precluding exposure of persons to UV-C radiation when sterilising small to medium-size objects. The use of UV-C lamps in air sterilisers would have a strong impact on public health and prevent exposure of the public to infectious aerosols. Currently, we do not know if SARS-CoV-2 in aerosols is inactivated by similar doses and the transferability of our results to viral aerosols might be limited. Nevertheless, our results may give a first indication on further use, even though dynamics and inactivation kinetics of virus in aerosols might differ. Hence, it is highly relevant and warranted to conduct studies to carefully determine UV-C doses necessary and sufficient for inactivation of SARS-CoV-2 in aerosols.

## Conclusions

We established the effectiveness of UV-C treatment against SARS-CoV-2 in a setting designed to simulate close-to-reality conditions of decontamination. The easy, rapid and chemical-free application of UV-C treatment to inactivate SARS-CoV-2 and its high efficacy demonstrates the potential of this technology in a broad range of possible settings.

## Supplementary Data

156000 Supplemental Material

## Supplementary Data

1301000 Supplemental Movie 1

## Supplementary Data

700000 Supplemental Movie 2
