# Erratum for Euro Surveill. 2021;26(42)

**DOI:** 10.2807/1560-7917.ES.2021.26.47.211125e

**Published:** 2021-11-25

**Authors:** Eurosurveillance editorial team

**Affiliations:** 1 European Centre for Disease Prevention and Control (ECDC), Stockholm, Sweden

In the article ‘Rapid, dose-dependent and efficient inactivation of surface dried SARS-CoV-2 by 254 nm UV-C irradiation’ by Ruetalo et al. published on 21 October 2021, the information about funding by Ministerium für Wissenschaft, Forschung und Kunst (MWK) Baden-Württemberg and University Hospital Tübingen was erroneously omitted in the original publication and added on 25 November 2021. The editors apologise for this mistake. 

